# Emergency Medicine Education and Research in Nepal: Challenges and Opportunities

**Published:** 2018-06-30

**Authors:** Anmol Purna Shrestha, Roshana Shrestha, Sanu Krishna Shrestha, Samjhana Basnet, Alok Pradhan

**Affiliations:** 1 Department of General Practise and Emergency Medicine, Kathmandu University School of Medical Sciences, Dhulikhel, Kavre, Nepal

## INTRODUCTION

Emergency medicine (EM) is a young but prestigious medical discipline worldwide.^1^ However, in Nepal, it is in preliminary phase.^2^ EM is not only restricted to urban emergency departments but also a multifaceted discipline.^3^ Several EM training modules are currently practiced fragmented with different curriculum and duration.^4–5^ Pre-hospital emergency medical services (EMS), hospitals, trauma centres, and public health are working in silos and most of them devoid of proper emergency facility.^2^ This brought us to the realization of an urgent need of bringing all the stakeholders together in a symposium like this.

The symposium was arranged into four different sessions as listed below:

1. To familiarize with the history and current state of EM from Global Emergency Medicine perspective.
2. To highlight the different clinical experiences and advancements in EM in Nepal.
3. To emphasize the importance and possibilities in EM education and research in Nepal.
4. To discuss the roadmap to develop and establish EM as a recognized medical speciality in Nepal.

The overall objectives of the symposium were to discuss the challenges faced by current Emergency Medicine providers and identify the opportunities for the future development and recognition in Nepal.

The most important current task for Nepal's emergency physicians of advocating for policies, programs, and funding to support further development of the specialty was realized. Rural and urban emergency service providers from academic and non-academic institutions, governmental/ non-governmental organizations and international medical institutions attended the symposium. General Practice (GP) residents, medical officers, medical students, interns and paramedics were among active participants.

## THE SYMPOSIUM REPORT

"Emergency Medicine Education and Research in Nepal: Challenges and Opportunities"

### 1. Global emergency medicine: past and present

A brief background of educational, research, clinical and community activities and engagements as a part of global health was discussed. The principles of global health partnership was discussed and the global health affiliation agreements between Medical College of Wisconsin (MCW), Patan Academy of Health Sciences (PAHS) and Dhulikhel Hospital (DH) was shared. Current status of health and EM in Nepalese context and focus on sustainable, locally and culturally sensitive improvement of EM were highlighted. Flashback to the history of EM in Nepal and DH, current Emergency department (ED) working scenarios and need of EMS in Nepal was also focused. A brief history of development of Doctor of Medicine (MD) in General Practice (GP) and Doctorate of Medicine (DM) in Emergency Medicine at Institute of Medicine (IOM), Nepal was explained. Challenges of developing and advancing EM into an independent medical specialty in North America over past 50 years were appreciated. National disaster preparedness and response strategy and the need of preparedness for both trauma and epidemiologic disasters in Nepal was emphasized in the session.

### 2. Emergency Medicine: heading towards efficiency

The importance of identifying red flags in pediatric emergencies that mimic simple pediatric illnesses was highlighted. Most of the fear surrounding caring for pediatric patients can be helped through pediatric emergency medicine education and training. Importance of point of care ultrasound (POCUS) in ED patient management was presented. Experiences were shared regarding helicopter emergency medical services (HEMS) in Nepal and its need taking into account the difficult terrains of Nepal and centralized health care services. Importance of standardized clinical handover of patients in all clinical settings was discussed.

### 3. Emergency Medicine: Education and research

The paradigm shift to Competency-based Medical Education- "Learning is not about time, but about outcomes" was emphasized. The discussion was centered around the current trend of shift of paradigm of competence and competence-based learning and assessment from being physician-centered to patient-centered and from being. teacher-driven to learner-driven. "Time being a resource, and not a framework", today's cutting edge medical education should be time-variable and competence-based. Discrepancies in distribution of the burden of diseases and concentration of healthcare services and clinical research in a global scenario was described. Importance of educating the community health care workers in emergency care by disseminating the experience of B.P. Koirala Institute of Health Sciences (BPKIHS) was shared. The scope of "In-situ simulation" to improve patient safety by identifying latent hazards and knowledge gaps of interdisciplinary staff involved in patient care and by strengthening their teamwork and technical skills was focused.

### 4. Panel discussion

The proceedings of the symposium were summarized and an insight into the future direction of EM in Nepal was further brainstormed in this session. Following future priorities were discussed:

- Advocacy for EM development
- Collaboration - National and International
- Education and training- Professional and academic development
- Equity in emergency care
- Emergency Care Research and
- Evidence Based Clinical service delivery.

The experts from various academic institutes of Nepal elaborated about why there has been lack of proper interest in this burning issue and how this could be addressed and improved. The GP doctors’ contribution to primary care and Emergency services to rural Nepal and urban emergency department, community education, pre-hospital care was also highlighted. Currently there are several diverse pathways in getting the Emergency medical degrees in Nepal and each is unique in its own way, however the need of uniform future program and the various pathways to reach the common definition of emergency physician were emphasized. The panel reached to a consensus that "General Practitioners’ Association of Nepal (GPAN) would take lead in this regard for betterment of overall emergency academia and clinical care in Nepal by forming a task force under its umbrella but embracing all current emergency physicians of all backgrounds. The best time to act by GPAN is NOW, otherwise it may be late.

### The feedback

An electronic feedback was collected from the participants. 70% of the participants responded that the symposium was relevant to their practice. The selection of topics was appropriate according to 66% participants.

About the respondents, 66.7% appreciated the venue of the symposium. 88.9% of participants found the oral presentations to be exceptional to appropriate. Majority of the participants appreciated the discussions, panel discussion and overall quality of activities. All the responders expressed that they would like to attend similar symposiums in the future. Themes suggested for future symposiums were:

- Challenges and Strategic management of Emergency medicine in Rural areas
- Emergency medicine-empowering ED
- Role of Paramedics as a physician assistant in life threatening condition of patient
- Standardizing emergency care throughout Nepal
- Recent advanced and updates in Emergency Medicine
- Working with stakeholders to advance the specialty of EM in Nepal

EMS education in Nepal, obstetric and gynaecological emergencies, pediatric emergencies, trauma systems and ATLS, ultrasonography in EM, high altitude medicine, EM and its role in NCDs were few areas suggested to be covered in future.

## WAY FORWARD

In this fast-paced world, ‘time is resource’ - as the practice of Emergency Medicine in Nepal is fragmented over places and modalities of delivery, it is high time that the stakeholders of Emergency Medicine reach a consensus and act with the same vision and one goal of uniform and advanced emergency care delivery. EM in Nepal is lagging far behind and we should be rapid in advocating and advancing EM in our part of world.

Collaboration between emergency physicians of all background (MD GP, Fellow EM, DM EM, and MD EM) and other stakeholders related to emergency care from different health facilities and organizations is essential to promote EM in all aspects and to create integrated networks of emergency care-from vision to action.
